# In Situ Atomic Reconstruction Engineering Modulating Graphene-Like MXene-Based Multifunctional Electromagnetic Devices Covering Multi-Spectrum

**DOI:** 10.1007/s40820-024-01391-8

**Published:** 2024-04-15

**Authors:** Ting-Ting Liu, Qi Zheng, Wen-Qiang Cao, Yu-Ze Wang, Min Zhang, Quan-Liang Zhao, Mao-Sheng Cao

**Affiliations:** 1 School of Materials Science and Engineering, Beijing Institute of Technology, Beijing, 100081 People’s Republic of China; 2 Department of Physics, Beijing Technology and Business University, Beijing, 100048 People’s Republic of China; 3 School of Mechanical and Materials Engineering, North China University of Technology, Beijing, 100144 People’s Republic of China

**Keywords:** Graphene-like MXene hybrids, Multi-spectral response, Multi-function antenna, Ultra-wideband bandpass filter, Electromagnetic device

## Abstract

With the diversified development of big data, detection and precision guidance technologies, electromagnetic (EM) functional materials and devices serving multiple spectrums have become a hot topic. Exploring the multispectral response of materials is a challenging and meaningful scientific question. In this study, MXene/TiO_2_ hybrids with tunable conduction loss and polarization relaxation are fabricated by in situ atomic reconstruction engineering. More importantly, MXene/TiO_2_ hybrids exhibit adjustable spectral responses in the GHz, infrared and visible spectrums, and several EM devices are constructed based on this. An antenna array provides excellent EM energy harvesting in multiple microwave bands, with |*S*_11_| up to − 63.2 dB, and can be tuned by the degree of bending. An ultra-wideband bandpass filter realizes a passband of about 5.4 GHz and effectively suppresses the transmission of EM signals in the stopband. An infrared stealth device has an emissivity of less than 0.2 in the infrared spectrum at wavelengths of 6–14 µm. This work can provide new inspiration for the design and development of multifunctional, multi-spectrum EM devices.

**Supplementary Information:**

The online version contains supplementary material available at 10.1007/s40820-024-01391-8.

## Highlights

- MXene/TiO_2_ hybrids are prepared by a simple calcination treatment, and their electromagnetic response is customized by in situ atomic reconstruction engineering.
- Based on the excellent electromagnetic response of MXene/TiO_2_ hybrids, a series of electromagnetic devices are constructed.
- Multi-spectrum stealth is realized covering visible-light, infrared radiation and GHz.

**Supplementary Information:**

The online version contains supplementary material available at 10.1007/s40820-024-01391-8.

## Graphical abstract

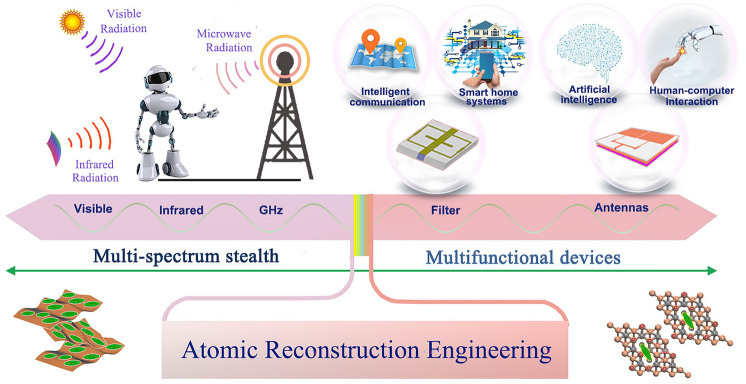

## Introduction

The booming development of artificial intelligence, big data, Internet of Things (IoTs) and other emerging science and technology industries has opened up a new epoch in human society in terms of intelligent life, information interaction and digital economy, and has also given electromagnetic (EM) functional materials and devices a new mission [1–4]. In this context, developing EM functional materials with efficient and broadband has become a hot research topic to protect human from EM radiation [5–8]. EM devices based on EM functional materials are the core components to realize the interconnection of everything and the convergence of data, and their research and development has now reached the most critical stage. In recent years, researchers have developed diverse EM devices in areas such as health monitoring, electronic communications and energy conversion, proving their unlimited application potential [9–12]. For example, using the excellent EM response properties of organic–inorganic hybrid perovskite microcrystals, Yan et al. [13] designed ultra-wideband bandpass filters with a passband up to nearly 8 GHz, which are capable of achieving high-quality transmission of EM wave signals in specific frequency bands. Zhao’s group assembled microwave-driven robots based on the microwave thermal effect, which can achieve controlled motion in a microwave environment [14]. The emergence of EM devices has given a strong impetus to cross-disciplinary development and has created the conditions for innovation in industry and manufacturing.

The research of EM devices is still in its infancy, and there are a lot of difficulties that need to be overcome and broken through by continuous researchers. EM functional materials, as the core of EM devices, have a decisive influence on the performance of the devices by their EM response characteristics. The modulation of the EM response relies on delicate preparation methods and fine structural tailoring on the micro-nano-scale [15–17]. However, many current works related to EM devices have neglected the role of material microstructure in regulating device performance, which is not conducive to performance optimization. More importantly, with the diversification of military investigation technology and the demand for practical applications, devices operating in a single frequency band are difficult to cope with multispectral detection methods, such as microwave, infrared light and visible-light detection. Therefore, the development of EM devices covering multiple spectrums is of strategic importance. However, it is a challenging scientific problem to realize compatible stealth based on the multispectral response of materials since the operating mechanisms and modes are different in each spectrum.

Two-dimensional (2D) transition metal carbides or nitrides (MXenes) have tunable electronic and dielectric properties due to their excellent conductivity, abundant surface functional groups and modifiable surfaces [18, 19]. This makes it a powerful candidate for the preparation of EM devices. Gogotsi's group prepared MXene patch antennas with high power radiation by pray coating from aqueous solution [20]. The antenna performance is comparable to copper patch antennas, demonstrating enormous potential in 5G applications. Li’s group constructed chemiresistive-type gas sensor using MXene/In_2_O_3_ heterostructure, which exhibits great response and selectivity to NH_3_ at room temperature [21]. Unfortunately, the current works still have limitations in terms of microstructure tailoring, multifunctional application and multispectral response.

Herein, we prepared Ti_3_C_2_T_*x*_/TiO_2_ hybrids with tailored EM response by different calcination temperatures. The hybrids show multispectral stealth capability in GHz, infrared and visible spectrums. At GHz, the sample possesses an optimum reflection loss (RL) of − 44.7 dB at GHz; at infrared band, the surface temperature of the sample is maintained at 38.6 °C under heating on a 50 °C hot plate; at visible-light band, the visible-light absorption rate can reach 78.2%. In addition, based on the multispectral response characteristics of MXene/TiO_2_ hybrids, several EM devices have been designed, including frequency-selective response antenna arrays, ultra-wideband bandpass (UWB) filters and low emissivity infrared stealth devices. The key parameters of the three devices are of the same order of magnitude or even better than those of the same type of EM devices previously reported (Table S1). This work is able to demonstrate the potential of MXene/TiO_2_ hybrid-based EM devices for applications in EM protection, wireless communication and information transmission.

## Experimental Sections

### Chemicals

Ti_3_AlC_2_ powder (300 mesh) was purchased from Shandong Xiyan New Material Technology Co., Ltd. LiF was supplied by Shanghai Macklin Technology Ltd. Hydrochloric acid (35 wt%) was obtained from Beijing Chemical Plant. All reagents were of analytical grade and used without further purification.

### Synthesis of MXene/TiO_2_ Hybrids

Firstly, 0.5 g of Ti_3_AlC_2_ (300 mesh) was added gradually to a mixture of 10 mL 9 M HCl and 0.5 g LiF and stirred at 48 °C for 48 h. Then, the product was washed repeatedly with deionized water until pH ≥ 6. The precipitate was then centrifuged at 3500 rpm for 10 min, and the resulting supernatant was centrifuged at 7000 rpm for 10 min. The finally obtained supernatant (Ti_3_C_2_T_x_ nanosheets colloidal solution) was freeze-dried to obtain MXene nanosheets. Finally, the nanosheets were subjected to calcination treatment at 200, 300, 400 and 500 °C for 2 h in an argon atmosphere at a heating rate of 5 °C min^−1^, corresponding to the sample names MT-2, MT-3, MT-4 and MT-5.

### Characterization

The composition and crystal structure of the samples were characterized by X-ray diffraction (XRD) on X 'Pert PRO system with a CuKα radiation source. Transmission electron microscopy (TEM) and high-resolution TEM (HRTEM) images were obtained by JEM-2100TEM system. The scanning electron microscopy (SEM) images were identified on Hitachi S-4800. Atomic force microscopy (AFM) images were obtained on a Veeco Dimension Fast Scan system. X-ray photoelectron spectroscopy (XPS) analysis was carried using an X-ray photoelectron spectrometer (PHI Quantera). The optoelectronic properties were characterized by UV–Vis diffuse reflectance spectroscopy. The photocurrent density curve of the sample was recorded under the cooperative effect of an electrochemical workstation (CHI 660e) and a periodic visible-light irradiator. The EM parameters were measured on a vector network (VNA, Anritsu 37269D). Typically, the samples were mixed with paraffin in a mass ratio of 3:7 and were pressed into a toroidal shape (*Φ*_outer_ = 7.03 mm; *Φ*_in_ = 3.00 mm) with thicknesses of ∼2 mm for measurement.

## Results and Discussion

### Morphology and Structural Characterizations

**Fig. 1:**
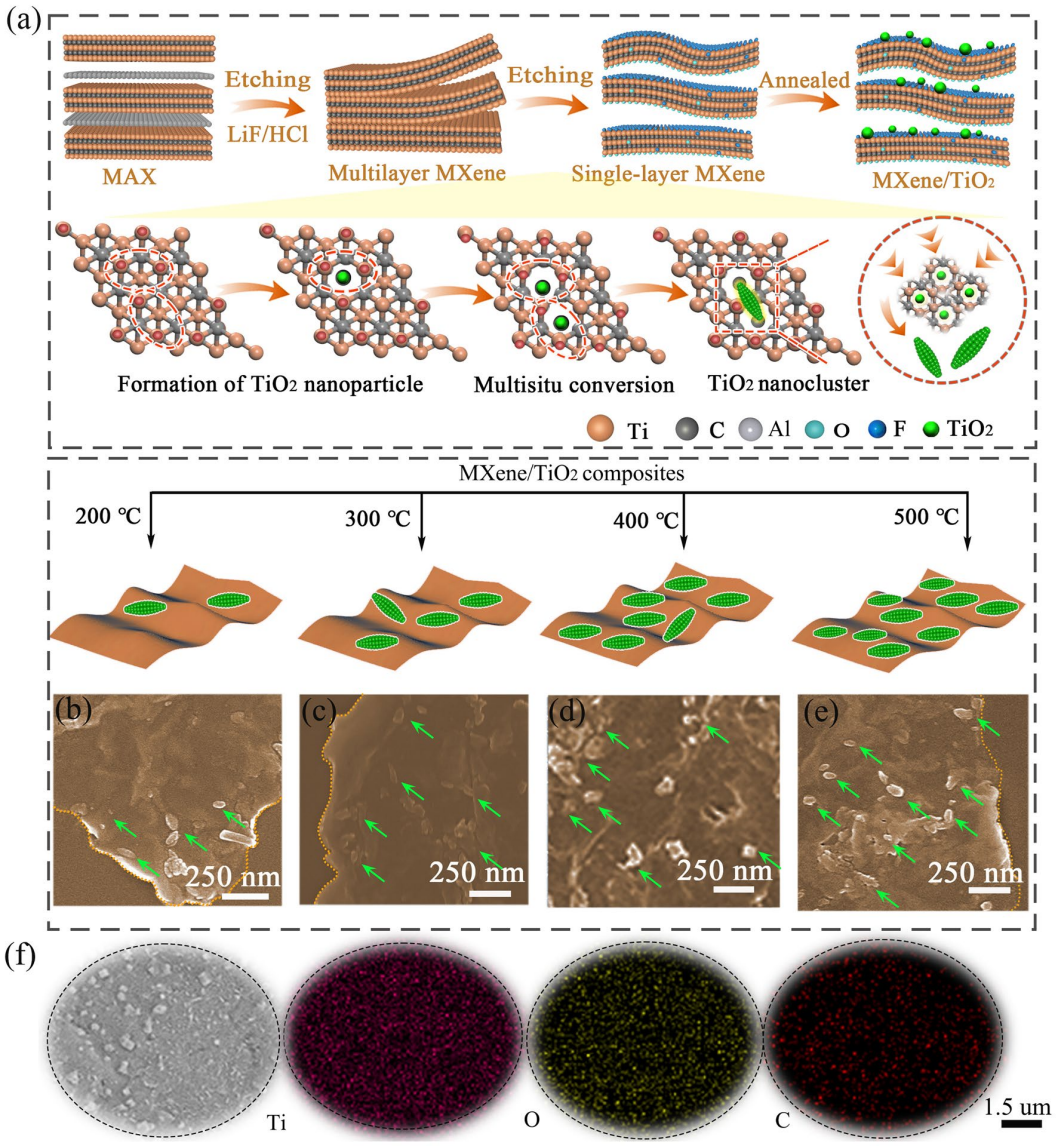
Fabrication and microstructure models of MXene/TiO_2_ hybrids. **a** Structural evolution process of the MXene/TiO_2_ hybrids. **b**–**e** Morphology of MT-2, MT-3, MT-4 and MT-5. **f** EDS mappings of MT-5

The schematic of in situ atomic reconstruction engineering for the preparation of MXene/TiO_2_ hybrids is shown in Fig. 1a. Ti_3_AlC_2_ MAX undergoes etching with lithium fluoride and dilute hydrochloric acid, transforming into monolayer Ti_3_C_2_T_*x*_ MXene and forming a large number of oxygen-containing functional groups on their surface. During the calcination process, these functional groups act as confine sites, promoting the transformation of Ti atoms into TiO_2_ nanoclusters [22]. The limited oxygen atoms in MXenes cause TiO_2_ nanoclusters to stop growing after reaching a certain scale, thus avoiding excessive aggregation of TiO_2_. The morphology of MXene/TiO_2_ hybrids can be tailored by the calcination temperatures. With the increase of calcination temperature, Ti atoms are thermally activated and the reaction rate increases, resulting in an increased coupling probability between Ti atoms and oxygen-containing functional groups. As shown in Fig. 1b–e, this leads the number of TiO_2_ nanoclusters on the surface of MXene increasing, indicating the regulatory effect of temperature on TiO_2_ crystal growth. EDS mapping images of Ti, O, and C elements demonstrate the uniform distribution of TiO_2_ nanoclusters on the MXene surface (Fig. 1f). The evolution of MXene/TiO_2_ hybrid structure is further characterized by XRD (Fig. S1a). In addition to the characteristic peaks of MXene located at 6.5° and 60.7°, the characteristic peaks of rutile-phase TiO_2_ are also present in the hybrids, and the intensity of the peaks increase with the calcination temperature, implying better crystallinity of TiO_2_ and stronger oxidation of MXene [23].

The surface elemental composition of the MXene/TiO_2_ hybrids was explored by XPS. In the survey XPS spectra, the MXene/TiO_2_ hybrids contain C, Ti, O and F elements, implying the presence of O-containing groups and F-containing groups in the MXene/TiO_2_ hybrids after etching and calcination process (Fig. S1b). The high-resolution XPS narrow spectra of Ti, C and O elements are shown in Fig. S1c–e. The high-resolution Ti 2*p* XPS spectra can be fitted to six peaks, of which those located at 458.9 eV (Ti 2*p*_3/2_) and 464.6 eV (Ti 2*p*_1/2_) are attributed to Ti–O, demonstrating the formation of TiO_2_ (Fig. S1c) [24]. The peaks at 454.7 and 461.1 eV are due to Ti–C, and the peaks at 457.3 and 462.7 eV are due to Ti^3+^ [25]. The four peaks located at 281.6, 284.8, 286.6 and 288.4 eV in the high-resolution C 1 s XPS spectra correspond to the C–Ti–Tx, C=C, C–O and O–C=O, respectively (Fig. S1d) [26]. The peaks located at 530.1 and 532.1 eV in the high-resolution O 1 s XPS spectra can be attributed to Ti–O–Ti and –OH, respectively (Fig. S1e) [27]. The above results further confirm the presence of TiO_2_ on the surface of MXene.

**Fig. 2:**
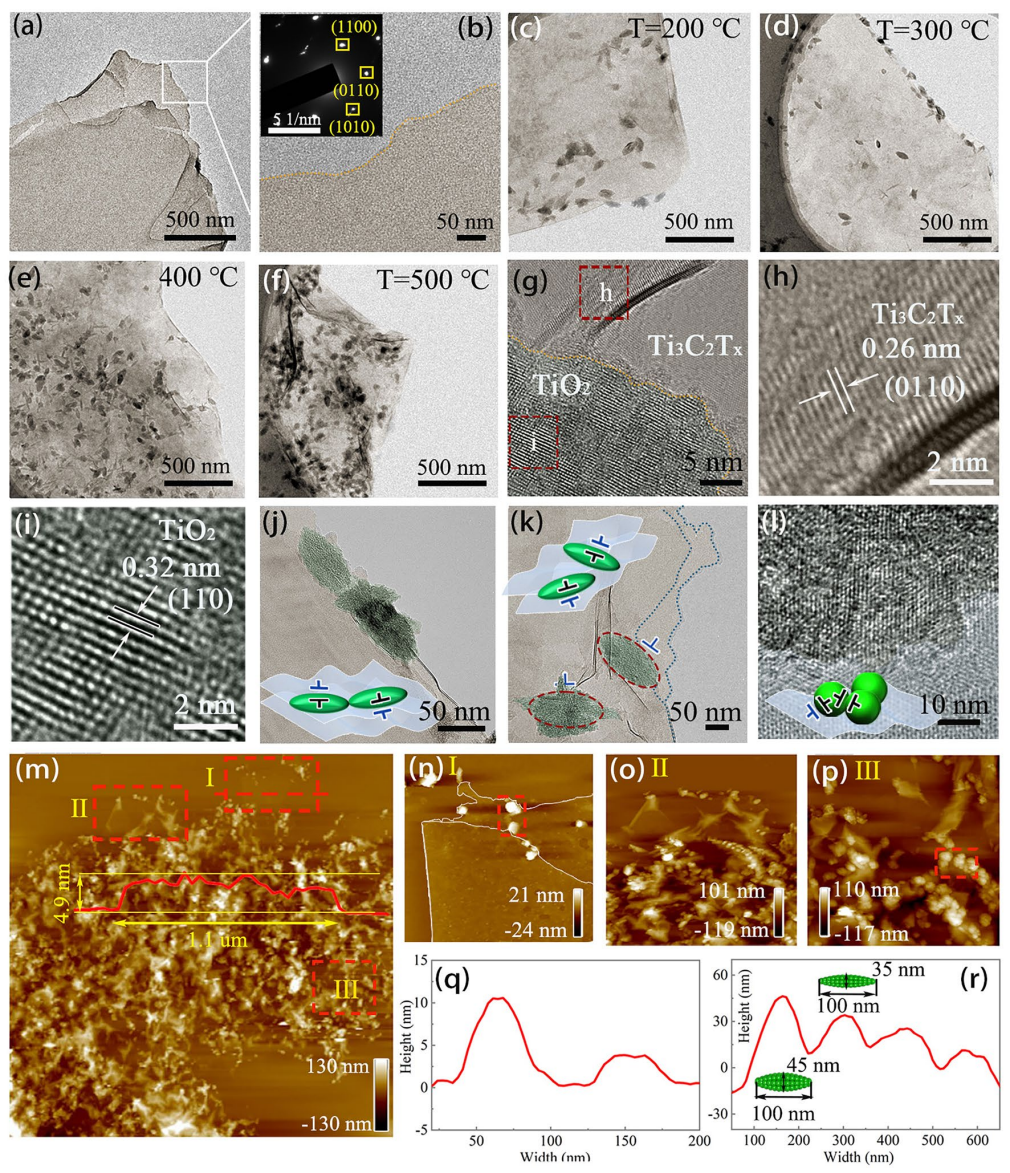
Microstructure characterizations of MXene and MXene/TiO_2_ hybrids. TEM image of **a, b** Ti_3_C_2_T_x_ MXene, **c** MT-2, **d** MT-3, **e** MT-4 and **f** MT-5. **g**–**i** HRTEM images of MT-5. **j**–**l** Interfaces in MXene/TiO_2_ hybrids. **m** AFM image of MT-5. **n**–**p** Enlarged view of region I, II and III in **m**, respectively. **q, r** The height distribution along the dashed line in **n** and **p**, respectively

TEM was used to further reveal the microstructure of the MXene/TiO_2_ hybrids. The MXene nanosheets exhibit an ultrathin multilayer structure, and the selected area diffraction (SAED) image in the insert indicates that MXene belongs to the hexagonal crystal system (Fig. 2a, b). After the calcination treatment, TiO_2_ nanoclusters with the particle size between 60 and 115 nm are uniformly distributed on the surface of MXene, and the number increases with rising temperature (Figs. 2c-f and S2). The high-resolution TEM (HRTEM) image of MXene/TiO_2_ hybrids shows that the lattice spacings of the two phases are 0.26 and 0.32 nm, corresponding to the (0110) crystal plane of MXene and the (110) crystal plane of TiO_2_, respectively (Fig. 2 g–i). In addition, the presence of irregular lattice stripes indicates a large number of structural defects in the MXene/TiO_2_ hybrids. Meanwhile, the introduction of TiO_2_ nanoclusters promotes the formation of interfaces, including interfaces between TiO_2_ nanoclusters and between TiO_2_ nanoclusters and MXene (Fig. 2j–l). The existence of defects and interfaces demonstrates the effectiveness of in situ atomic reconstruction engineering for tailoring MXene microstructures, enhancing the EM response characteristics of MXene/TiO_2_ hybrids. The scale of the hybrids was characterized by AFM (Fig. 2m–p). In Fig. 2m, the two-phase structure of the hybrids is well defined, and the edge sites of MXene are exposed in regions I and II. The monolayer height of the bilayered MXene is about 2.45 nm, which is close to what was previously reported (Fig. 2n) [28]. In addition, the size of TiO_2_ nanoclusters at different regions is detected. As shown in Fig. 2q, r, the TiO_2_ nanoclusters in region I are 5 nm in height and ~ 50 nm in width, and the TiO_2_ nanoclusters in region III are more well-crystallized, 30–45 nm in height and ~ 100 nm in width. The construction of micro-nanostructures by combining TiO_2_ nanoclusters of different sizes with MXene is beneficial for achieving superior EM functions.

### EM Response and Loss of MXene/TiO_2_ Hybrids

**Fig. 3:**
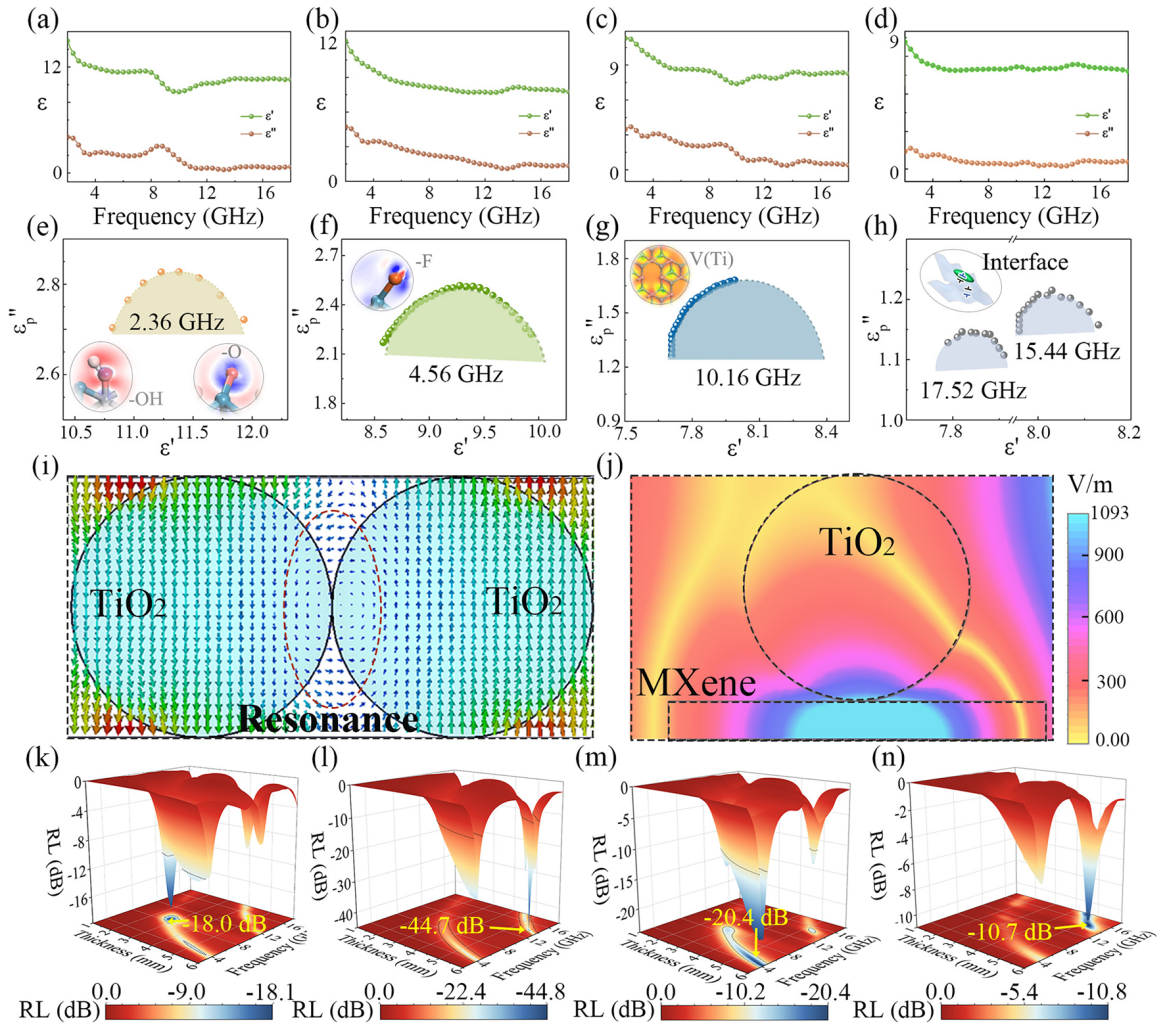
EM response and microwave absorption performance of MXene/TiO_2_ hybrids. Complex permittivity of **a** MT-2, **b** MT-3, **c** MT-4 and **d** MT-5. **e**–**h** Cole–Cole plots of MT-3. The inserts are the charge difference density images of MT-3 with –OH, –O, and –F and that with Ti-vacancy defect. **i, j** Simulation images for E-field between two TiO_2_ nanoparticles, as well as between TiO_2_ nanoparticle and MXene. RL values of **k** MT-2, **l** MT-3, **m** MT-4 and **n** MT-5 versus frequency and thickness

The EM response characteristics of MXene/TiO_2_ hybrids are explored by complex permittivity (Fig. 3a–d). With incremental frequency, the real permittivity (*ε'*) first decreases and then tends to level off. In addition, the *ε'* decreases as the calcination temperature increases. The imaginary permittivity (*ε"*) reflects the absorption capacity of the material to EM waves, which is dominated by conduction loss (*ε*_*c*_*"*) and polarization relaxation loss (*ε*_*p*_*"*) [29]. The *ε*_*c*_*"* values are lower when the calcination temperature is higher, which means that the generation of TiO_2_ will disrupt the electron transport channel on the surface of MXene, reducing the conductivity (Fig. S3a, b) [30]. The *ε*_*p*_*"* curves of all samples have multiple fluctuation peaks, indicating the presence of multiple polarization relaxation (Fig. S3c). To further investigate polarization relaxation, the Cole–Cole plots of MT-3 are plotted. According to Debye’s theory, semicircles in the Cole–Cole plots indicate occurring polarization relaxations in the materials [31]. As shown in Figs. 3e–h and S4, semicircles can be observed at the frequencies of 2.36, 4.56, 10.16, 15.44 and 17.52 GHz, indicating the presence of multiple relaxation mechanisms in MT-3 caused by functional groups, defects and interfaces (the inserts in Fig. 3e–h). MXenes produce functional groups and defects during etching and calcination, and their asymmetric charge density distribution forms dipoles, whose rotation lags behind the changes in the alternating EM field, resulting in the attenuation of EM waves [32]. In addition to this, interface polarization can be formed between interfaces within the material, which also contributes to the relaxation loss. As shown in Fig. 3i, j, the electric field is not uniform at the interface between TiO_2_ nanoclusters, nor at the interface between TiO_2_ nanocluster and MXene nanosheets. The difference in charge capacity between the two sides of the interface creates interface dipoles, resulting in fluctuations in *ε*_*p*_*"* [33]. In summary, in situ atomic reconstruction engineering effectively regulates the EM response of MXenes.

Based on their excellent EM response characteristics, MXene/TiO_2_ hybrids exhibit good EM wave absorption ability (Fig. 3k–n). The calcination temperature flexibly adjusted the performance and the optimal RL for the four samples reaches − 18.0, − 44.7, − 20.4 and − 10.7 dB, respectively. Their effective absorption bandwidths are 2.80, 3.84, 2.00 and 0.56 GHz, respectively. MT-3 is superior to other samples because it has better impedance matching. As shown in Fig. S5, the impedance matching values of MT-3 cover the maximum area in the range of 0.8–1.2, indicating that more EM waves can enter the material [34–36].

### Multifunctional EM Devices

#### Multifunctional Antennas

**Fig. 4:**
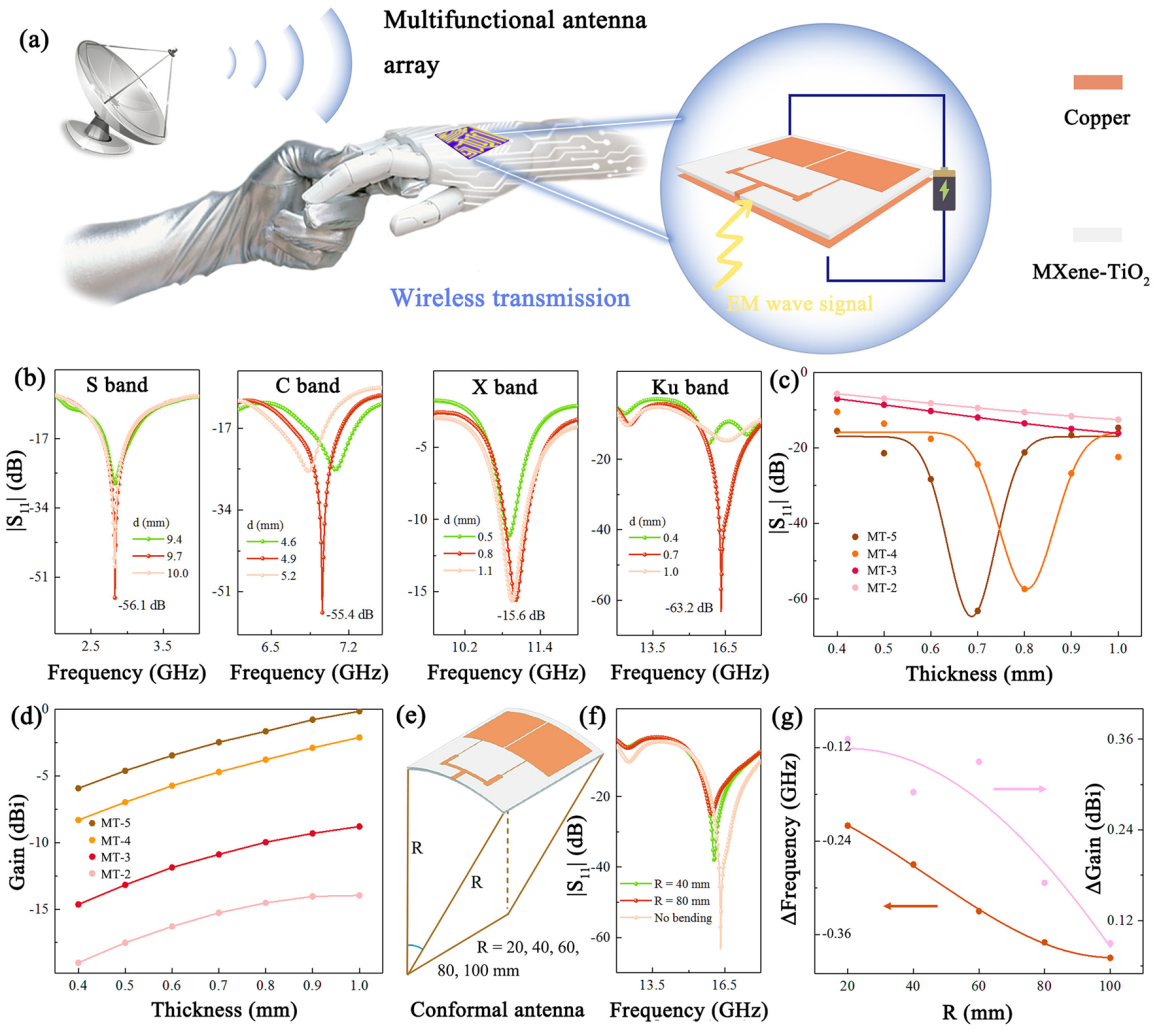
A multifunctional microstrip antenna array constructed by MXene/TiO_2_ hybrids. **a** Structure of the antenna array. **b** *|S*_11_*|* curves versus frequency. **c** Minimum *|S*_11_*|* values at different substrate thicknesses. **d** The maximum gains of MXene/TiO_2_ hybrids antenna arrays. **e** Schematic diagram of conformal antenna array. **f** *|S*_11_*|* curves at different degrees of bending. **g** Offset of center frequency and increment of gain (relative to unbending antenna array)

Considering the typical EM response characteristics, it is conceivable that Ti_3_C_2_T_x_/TiO_2_ has significant application prospects in EM devices for wireless communications. The emergence of microstrip patch antennas has been a catalyst for advancing microwave integration technology and pioneering new fabrication methods. These antennas have shown great potential for applications in a range of fields including satellite communications, navigation, telemetry, remote control, weapon fusing, medical devices and wearable devices [37–39]. To further exemplify the potential application of MXene/TiO_2_ in this technology area, we designed a conceptualized multifunctional 1 × 2 antenna array, which cleverly achieves the functional integration of EM stealth and signal transmission (Fig. 4a). Due to the intrinsic EM characteristics of MXene/TiO_2_ hybrids, a portion of the EM waves are received by the antenna and converted into electrical energy. Meanwhile, another portion of EM waves will be converted into thermal energy. Therefore, the antenna not only achieves signal transmission, but also avoids pollution caused by residual EM waves. This antenna array consists of three distinct components, each contributing to the harmonious coordination of its multifaceted functions. From top to bottom, these components include a copper radiating patch, a MXene/TiO_2_ dielectric substrate and a copper grounding plate.

The adjustment of the antenna array size and dielectric substrate thickness achieves precise control of the antenna array operating band, as shown in Figs. 4b and S6. When MT-5 is employed as the substrate, the resonant frequency of the antenna array can be precisely tuned to multiple frequency bands covering the S, C, X and Ku bands. Notably, the peak value of the return loss (*|S*_11_*|*) exceeds − 10 dB in all these bands, thus demonstrating the effective signal reception of the antenna array in the specified frequency range [40]. For example, in the Ku band, the *|S*_11_*|* value can be minimized to a remarkable − 63.2 dB when the substrate thickness is only 0.7 mm. This unique characteristic of operating in a specific frequency range is important for EM stealth applications. The structural configuration and EM characteristics of the substrate are key factors in manipulating the performance of the antenna array. The EM coupling between the substrate and the radiating patch has an impact on the radiation characteristics and frequency response of the latter. In addition, the thickness and EM response of the substrate have a significant effect on various parameters such as operating frequency, impedance matching, radiation direction, gain and bandwidth of the antenna array. It can be seen that the return loss deteriorates when the thickness of the MT-5 substrate is away from 0.7 mm (Fig. 4c). As the substrate thickens, the impedance of the antenna increases, leading to deterioration of *|S*_11_*|*. Conversely, a thinner substrate results in lower impedance. In addition, the construction of the conduction network also has an impact on the performance. For example, the *|S*_11_*|* value of the MT-2 antenna is only − 12.6 dB for a substrate thickness of 1.0 mm. This phenomenon is attributed to the improvement of the internal conduction network of MT-2, which leads to an increase in the reflection of EM waves. The EM properties of the substrate can also have an effect on the gain of the antenna array. Figures 4d and S7 show that the maximum gain of all four antenna arrays increases as the substrate thickness increases. In particular, the MT-5 antenna achieves the highest gain due to its moderate dielectric loss. However, it is worth noting that while higher loss dielectric substrates may worsen return loss and reduce gain, they simultaneously widen the impedance bandwidth (*|S*_11_*|*≤ − 10 dB). When the substrate thickness is 1.0 mm, the impedance bandwidth of the MT-3 and MT-2 antennas reaches an impressive 6.0 GHz, which comprehensively covers the entire Ku band (Fig. S8). This phenomenon is related to the interaction between the improved dielectric loss and the enhanced conductivity network. The combined effect of the two reduces the quality factor of the antenna, which is negatively correlated with the impedance bandwidth [41]. In summary, the *|S*_11_*|*, gain, and bandwidth in antenna arrays can be precisely controlled by adjusting the structure and EM characteristics of the substrates. Figure S9a-c, Movie S1 and S2 provide vivid depictions of the surface current distribution on the radiation patch, the E-field and the H-field distribution on the substrate. The current emanates from the feeder and flows along the narrow edge of the copper sheet, subsequently creating a strong displacement current distribution between the patch and the grounding plate.

The performance of the antenna array when subjected to bending was explored, where the model is covered with a cylindrical surface of radius R. A decrease in the value of R corresponds to an increase in the curvature of the antenna array, as shown in Fig. 4e. Even in this conformal state, the *|S*_11_*|* of the antenna array exhibits a commendably stable level, as shown in Fig. 4f. Along with the increase in degree of bending, the center frequency of the MT-5 antenna is shifted to a lower frequency and at the same time the increment of gain increases (Fig. 4g). Figure S9d-i shows that in a bent state, the surface current intensity of the antenna increases, while the E-field and H-field change from uniform distribution on the dielectric substrate to concentrated distribution in certain areas. This has led to a certain degree of deterioration in the return loss. The high sensitivity of antenna arrays under bending and deformation enables them to empower wearable devices with wireless communication, information transfer, deformation sensing, and more. In addition, proper dielectric loss gives these low gain antennas a wide directional range while minimizing radiation to the human body.

#### UWB Bandpass Filter

**Fig. 5:**
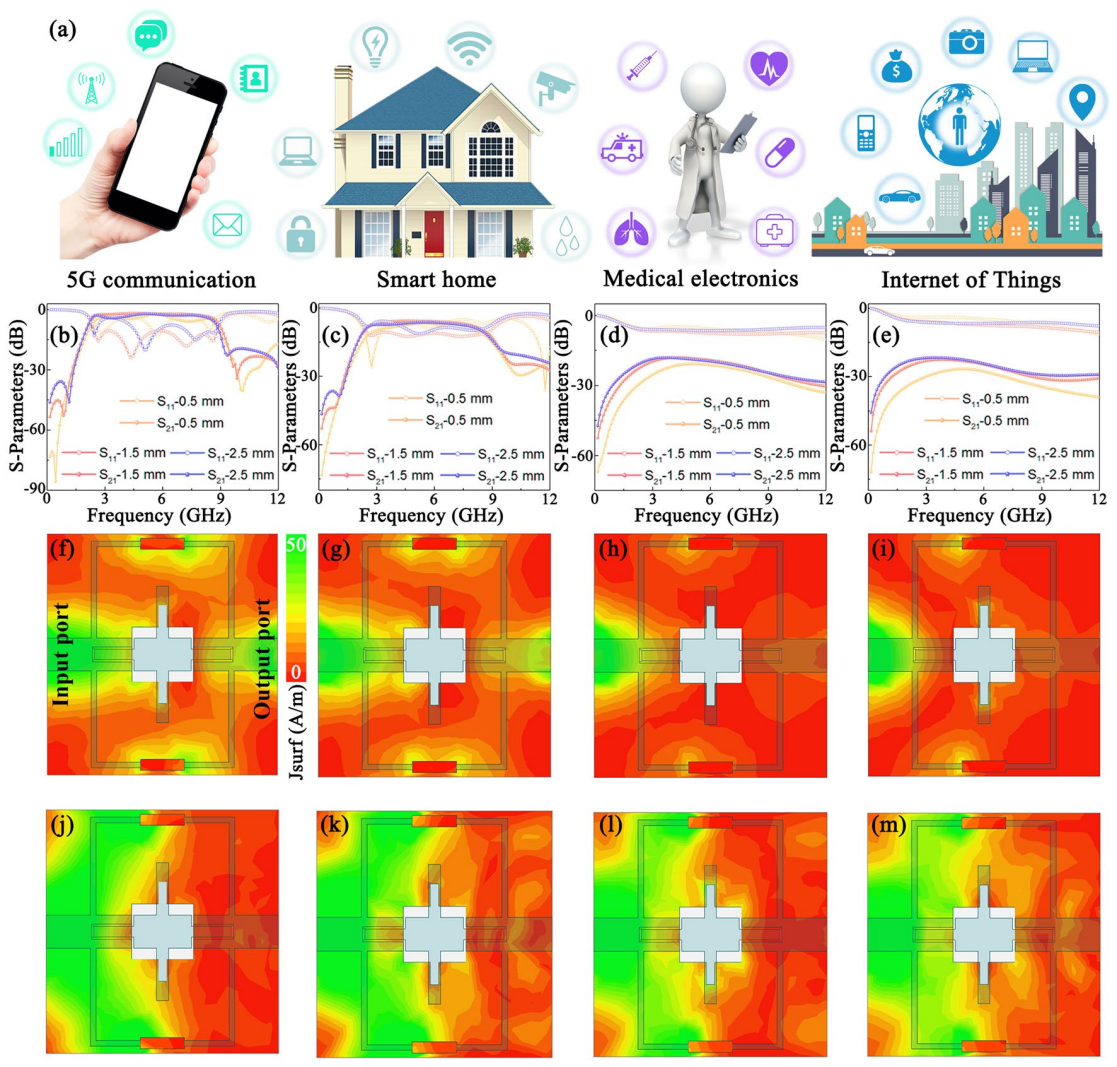
An UWB bandpass filter constructed by MXene/TiO_2_ hybrids. **a** Application scenarios of UWB bandpass filters. |*S*_11_| and |*S*_21_| curves of** b** MT-5, **c** MT-4, **d** MT-3, and **e** MT-2 bandpass filters. **f**–**i** Surface current distributions in passband. **j**–**m** Surface current distributions in stopband

A bandpass filter is an electronic component that allows EM waves to pass within a specific frequency band while preventing their transmission outside the specified range. They have a wide range of applications in different fields, including 5G communications, smart home systems, medical electronics, IoTs, security monitoring and automotive electronics, as shown in Fig. 5a [42–44]. A UWB bandpass filter is constructed based on the superior EM response of MXene/TiO_2_. Figure S10 shows the structure of the filter, including a copper transmission line topology on the upper surface, a MXene/TiO_2_ dielectric substrate in the center, and a copper grounding plate on the lower surface. Accurate tailoring of the EM response of MXene/TiO_2_ is critical for manipulating the performance of bandpass filters. The MT-5 bandpass filter offers the best overall performance, including high return loss (*|S*_11_*|*, ≥ 10 dB in the passband), low insertion loss (|*S*_21_|, up to 1.82 dB), a wide passband (5.44 GHz, 2.55–7.99 GHz, and covering virtually the entire S and C bands), and strong suppression outside the band (53.4 dB) (Fig. 5b). These excellent properties can be attributed to the low dielectric loss of the MT-5 substrate. However, the MT-4 bandpass filter has a power attenuation of more than 44% due to its higher conductivity and dielectric loss, resulting in a larger insertion loss (*|S*_21_*|*> 5 dB) in the 2–8 GHz range, which prevents the formation of an effective passband as shown in Fig. 5c. For the MT-3 and MT-2 bandpass filters, the tight conductivity network formed by MXene/TiO_2_ attenuated most of the EM waves, resulting in the weakening of the signal strength, as shown in Fig. 5d, e. The material, thickness of the dielectric substrate and metal patch jointly affect the radiation characteristics and impedance matching of the filter. Among them, the thickness of the dielectric substrate has a significant impact on the S-parameter of the filter. An increase in thickness will cause the center frequency of the passband to shift slightly toward lower frequencies, because the thickness affects the phase of microwave propagation. In addition, excessive thickness will increase the quality factor, thereby reducing the passband bandwidth. Within an appropriate range, increasing the thickness can enhance the return loss within the passband, but at the same time, the insertion loss within the stopband will also be smaller. Therefore, in order to achieve good overall performance, it is necessary to comprehensively regulate the permittivity, dielectric loss and thickness of the dielectric substrate.

The surface current distribution can reflect dynamic performance of bandpass filters. The surface current distribution of the MT-5 bandpass filter in the passband is shown in Fig. 5f, indicating that the currents are mainly concentrated in the transmission-line topology, which implies that a large portion of the EM energy can be efficiently moved from the input port to the output port. Movie S3 vividly depicts the dynamic evolution of the surface currents in the passband. As the phase angle rises, the traveling wave propagates from the input to the output, facilitating the movement of the surface current from the input to the output. This process confirms the commendable performance of the MT-5 bandpass filter in the passband. However, the reduced current density of the MT-4 filter at the output port means that some of the EM energy is blocked (Fig. 5g). Moreover, the MT-3 and MT-2 filters do not observe the presence of current at the output port, indicating that no effective passband is formed (Fig. 5h, i).

Figure 5j depicts the current distribution of the MT-5 bandpass filter in the stopband. The current is mainly distributed near the input port, indicating that the transmission of EM energy is effectively suppressed. The periodic dynamics of the current in the resistance band is shown in Movie S4, in which EM waves emitted by a signal source encounter obstructions at the input port and are reflected. These reflected waves are then superimposed on successive incident EM waves, generating standing waves and causing current to accumulate near the input port. Combined with the results of insertion loss and return loss, the excellent performance of the MT-5 bandpass filter in suppressing the EM energy in the stopband can be further confirmed. On the contrary, the current of MT-2, MT-3, and MT-4 filters gradually migrates towards the transmission-line topology, indicating poor blocking effect on the EM energy (Fig. 5k–m). In summary, the tuning of the conductive network and polarization relaxation by varying the calcination temperature enables the precise tailoring of the EM response of MXene/TiO_2_, which can adjust the performance of the bandpass filters.

### Multi-Spectrum Stealth-Infrared and Visible Light

**Fig. 6:**
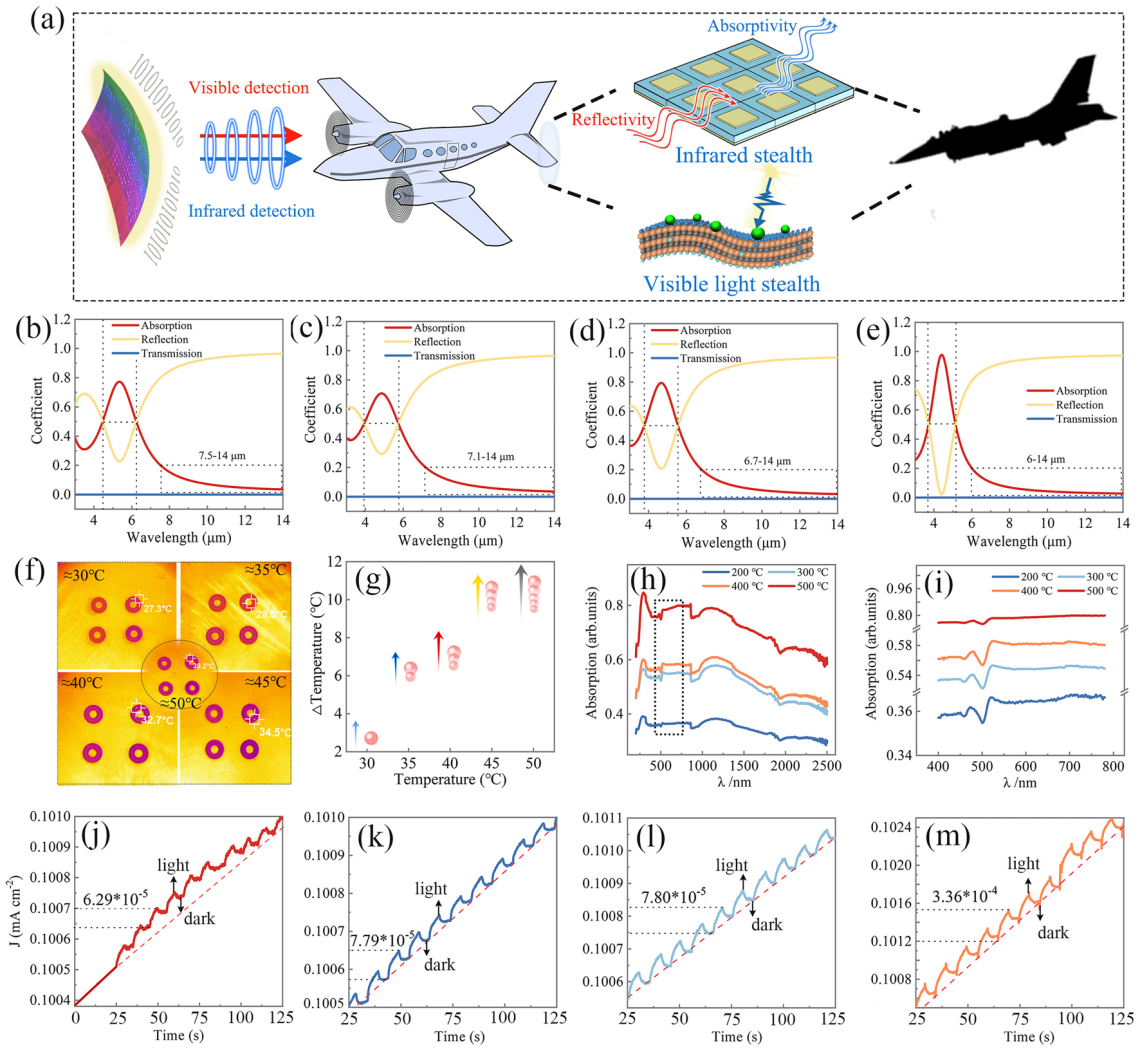
**a** Infrared and visible-light response characteristics of MXene/TiO_2_ hybrids. **a** Schematic of infrared stealth and visible-light stealth. The power coefficient of **b** MT-2, **c** MT-3, **d** MT-4 and **e** MT-5 infrared stealth device. **f** Thermal infrared images. **g** Temperature increments under heating conditions. **h** UV–Vis absorption curves of MXene/TiO_2_ hybrids. **i** Enlarged view of the localized area in **h**. Current density of **j** MT-2, **k** MT-3, **l** MT-4 and **m** MT-5

In the military domain, faced with increasingly complex and sophisticated EM monitoring systems, multispectral stealth technology has become a key focus of research as a countermeasure, providing a high degree of secrecy for military operations, as shown in Fig. 6a. Against this backdrop, it has become crucial to reduce the infrared radiation characteristics of the target through the development of infrared stealth materials to evade locating, tracking and guiding attacks by detectors [45, 46]. To evaluate the potential applications of MXene/TiO_2_ hybrids in the field of infrared stealth, an innovative patterned infrared stealth device is constructed, as shown in Fig. S11. The stealth device unit consists of a copper metal resonant unit, a lossy MXene/TiO_2_ hybrids dielectric layer, and a copper metal backplate. The working principle is that the EM wave incident on the structure triggers the reverse current in the two layers of metal plates, which induces the interaction between the induced electric field and the external electric field to produce an induced magnetic field. When coupled with an external magnetic field, a resonant response of EM waves is generated. Therefore, the degree of matching between the induced magnetic field and the external magnetic field can be adjusted through the material design of the dielectric layer, thereby modulating the infrared stealth performance of the material.

The EM response of MXene/TiO_2_ is flexibly customized by in situ atomic reconstruction engineering, the performance of infrared cloaking devices can be accurately controlled. Figure 6b–e shows the performance curves of the infrared stealth device, which reveals the relationship of the performance on dielectric characteristics. Specifically, the absorption rate is negatively correlated with the calcination temperature. Among them, MT-5 shows the best infrared stealth performance, with an absorption rate of less than 0.2 over a range of 6–14 μm, a minimum emissivity of only 0.027, and an average emissivity as low as 0.08 (Fig. 6e and Table S2). This phenomenon can be traced to the fact that when the calcination temperature is 500 °C, the *ε*_*c*_*"* and *ε*_*p*_*"* of the material decreases significantly compared to the other samples, and the matching degree of the dielectric layer to the external magnetic field decreases, which increases the thermal reflection effect and effectively attenuates the EM wave emission. The infrared thermal imaging results are shown in Fig. 6f, which intuitively shows that the color of the covered area is significantly different from the surroundings at different ambient temperatures (30, 35, 40, 45 and 50 °C), strongly confirming the effective suppression of thermal radiation of the sample. The temperature above the sample is 38.4 °C after being placed on the heating platform at an ambient temperature of 50 °C, meaning that the excellent infrared stealth performance can be maintained at high temperatures (Fig. 6g).

Figure S12a-d shows the relationship of calcination temperature from thermal radiation suppression ability at the same ambient temperature. It was observed that the surface temperature of the sample decreased significantly with the increase of calcination temperature. Notably, MT-5 shows the greatest decrease in temperature difference, indicating that MT-5 has a better ability to suppress thermal radiation compared to the other samples. Thermal infrared images of MT-5 captured at every 2 min (from 1 to 7 min) are demonstrated in Fig. S12e-h. As compared to the ambient color, the hue of the MT-5 is brighter, further confirming the thermal infrared stealth potential of the MT-5. As clearly discerned, infrared stealth based on the outstanding EM performance of MXene/TiO_2_ has immense application potential in the fields of precision thermal management, thermal camouflage and selective thermal radiation.

Simultaneously, benefiting from the in situ atomic reconstruction engineering, MXene/TiO_2_ manifests excellent visible-light absorption capability. As shown in Fig. 6h, i and Table S3, all samples show effective absorption peaks in the visible-light region, and the average visible-light absorption rates are ranked as follows: MT-5 (78.2%) > MT-4 (57.7%) > MT-3 (54.5%) > MT-2 (36.3%). This can be attributed to the increase of calcination temperatures, which increases the amount of in situ transformed TiO_2_, thereby increasing the amount of photoactive sites, which enhances the light capture ability of the material. When the light incident on the surface of the material, the energy of the photon excites the internal electrons of the material, prompting their valence band to transition to the conduction band, and triggering the generation of photocurrent under the action of an applied voltage. Figure 6j–m shows the current density of 1 M KOH electrolyte at a 0.3 V constant bias, where MXene/TiO_2_ is recorded as an electrode with a Pt sheet electrode and a Hg/HgO reference electrode. The samples show repeatable response to the switching period of visible-light irradiation. As shown in Fig. 6j, during the initial 0–25-s period without light irradiation, no periodic change current was observed in the working electrode. When start of periodic light irradiation, the current density of all samples showed a periodic change, indicating that the MXene/TiO_2_ hybrid is photosensitive [47, 48]. More importantly, the MT-5 working electrode shows a greater amplitude of current density change than other samples throughout the cycle (Fig. 6j–m), indicating that MT-5 can be used in the construction of visible-light absorption devices. In summary, the multi-spectrum EM response of Ti_3_C_2_T_x_/TiO_2_ is realized by using in situ atomic reconstruction engineering, thus realizing multi-spectral stealth, and promoting its potential application prospects in camouflage, electronic communication, military defense and other fields.

## Conclusions

In conclusion, TiO_2_ nanoparticles are implanted in MXene through in situ atomic reconstruction engineering to modulate conduction loss and polarization relaxation. Excellent EM response characteristics endow MXene/TiO_2_ hybrids with favorable multispectral stealth capability, including GHz, infrared and visible light. In the GHz band, the optimal RL can reach − 44.7 dB, and the absorption performance can be manipulated by different calcination temperatures. In the infrared band, the surface temperature of MXene/TiO_2_ is only 38.4 °C after heating on a hot plate at 50 °C. In the visible-light band, the sample has a light absorption rate of 78.2%. Significantly, based on the multispectral response characteristics of the samples, three EM devices are designed, including an antenna array to realize the multi-frequency response in S, C, X and Ku band, an UWB bandpass filter with wide passband bandwidth of 5.44 GHz and an infrared stealth device with an average emissivity as low as 0.08. This work will lay a solid foundation for the research of EM functional materials and devices in multiple frequency spectrums and provide unique strategy for promoting the information construction in the future.

## Supplementary Information

Below is the link to the electronic supplementary material.

Supplementary file1 (AVI 10016 kb)

Supplementary file2 (AVI 7069 kb)

Supplementary file3 (MP4 91 kb)

Supplementary file4 (MP4 84 kb)

Supplementary file5 (PDF 1826 kb)
