# Supplementary material for: In Situ Atomic Reconstruction Engineering Modulating Graphene-Like MXene-Based Multifunctional Electromagnetic Devices Covering Multi-Spectrum
Source: Nanomicro Lett. 2024 Apr 15;16:173. doi: 10.1007/s40820-024-01391-8 (PMC11018580; doi:10.1007/s40820-024-01391-8)
Supplement: Supplementary file 5 — Supplementary file5 (PDF 1826 kb) [file 40820_2024_1391_MOESM5_ESM.pdf]

Supporting Information for

In-Situ Atomic Reconstruction Engineering Modulating Graphene-Like MXene-Based Multifunctional Electromagnetic Devices Covering Multi-Spectrum

Ting-Ting Liu[1, #], Qi Zheng[1, #], Wen-Qiang Cao[1], Yu-Ze Wang[1], Min Zhang[2], Quan-Liang Zhao[3] and Mao-Sheng Cao[1,*]

[1] School of Materials Science and Engineering, Beijing Institute of Technology, Beijing, 100081, P. R. China

[2] Department of Physics, Beijing Technology and Business University, Beijing 100048, P. R. China

[3] School of Mechanical and Materials Engineering, North China University of Technology, Beijing 100144, P. R. China

[#] Ting-Ting Liu and Qi Zheng contributed equally to this work.

*Corresponding author. E-mail: caomaosheng@bit.edu.cn (Mao-Sheng Cao)

Supplementary Figures and Tables

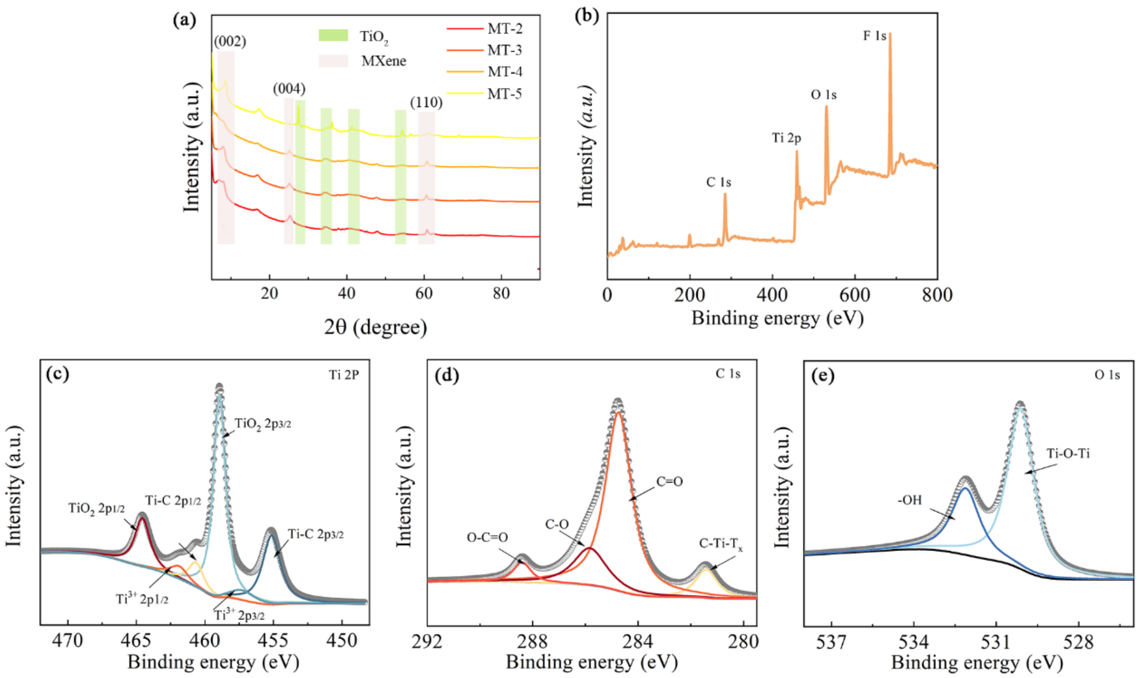

Fig. S1 a The XRD patterns of MT-2, MT-3, MT-4 and MT-5. **b** XPS wide scan (survey). High-resolution narrow scan on the **c** Ti 2*p*, C 1*s* **d**, and **e** O 1*s*, regions in the spectra

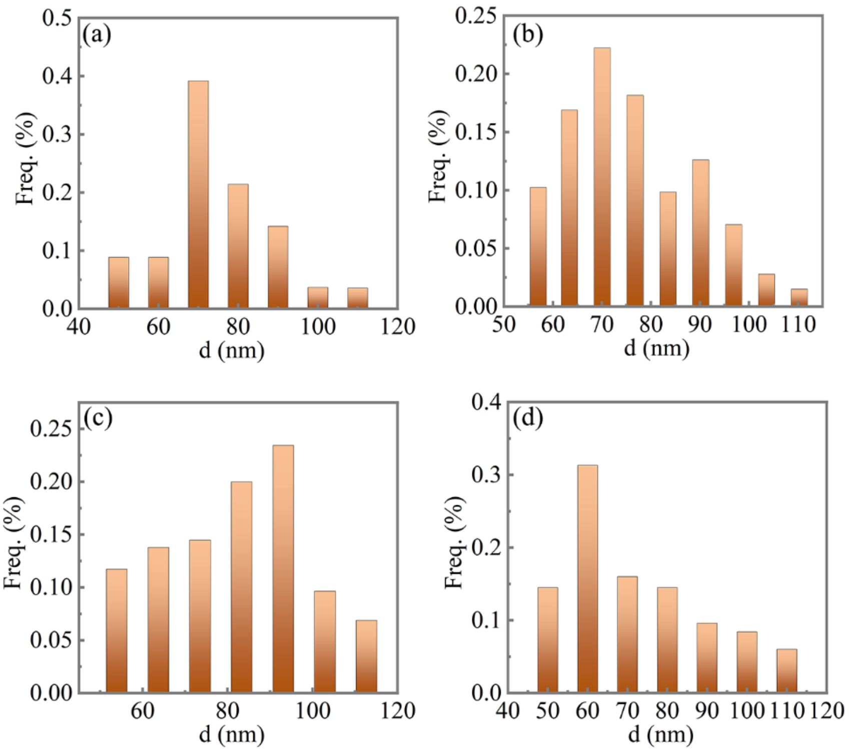

Fig. S2 TiO_2 particle size distribution map of **a** MT-2, **b** MT-3, **c** MT-4 and **d** MT-5

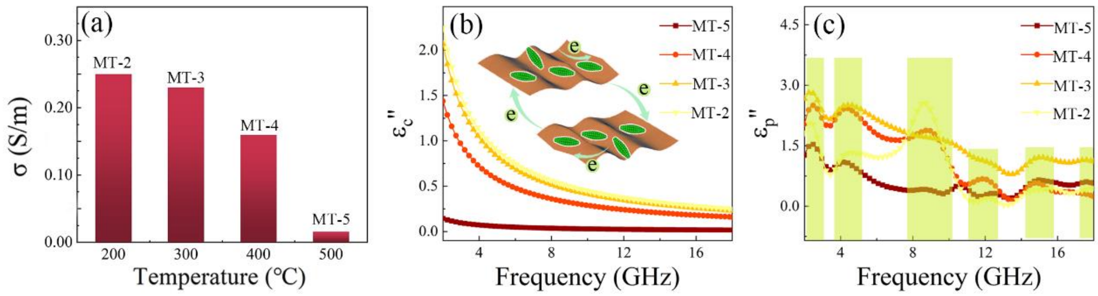

Fig. S3 **a** The conductivity of the MXene/TiO_2 hybrids with different calcination temperatures. **b** ε_c'' curves and **c** ε_p'' curves of MXene/TiO_2 hybrids versus frequency

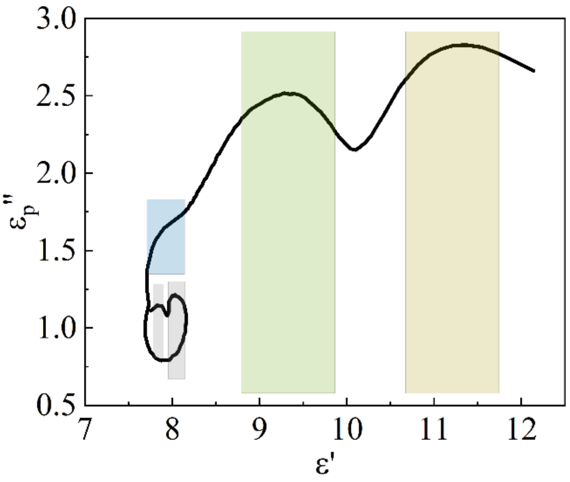

Fig. S4 Cole-Cole plots of MT-3

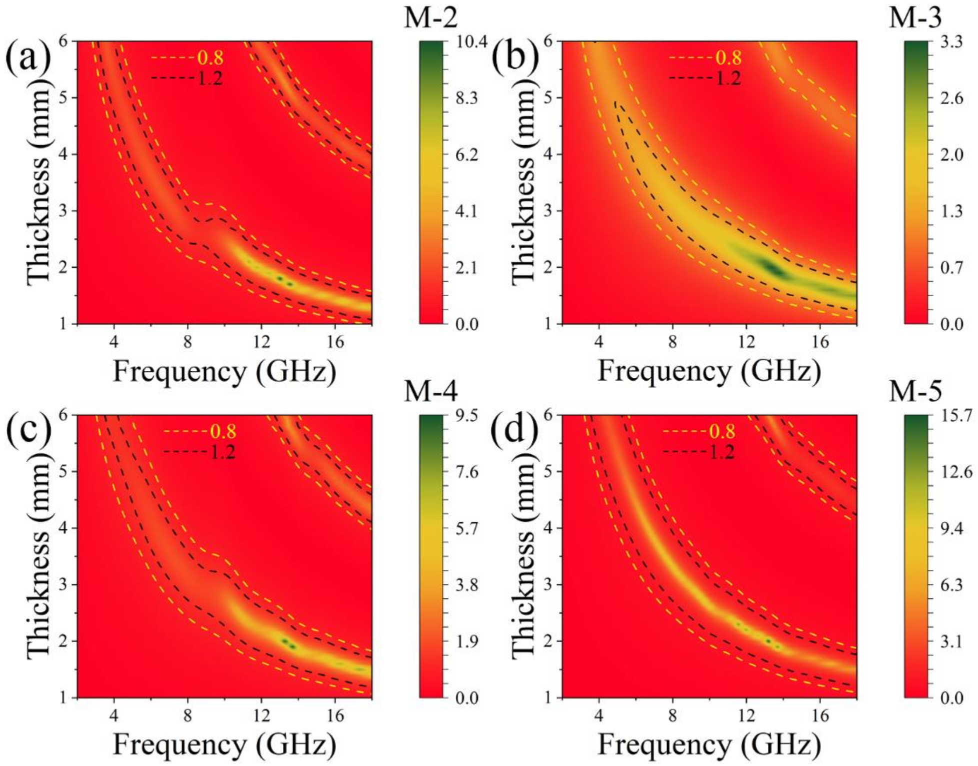

Fig. S5 Impedance matching of **a** MT-2, **b** MT-3, **c** MT-4 and **d** MT-5 versus frequency and thickness

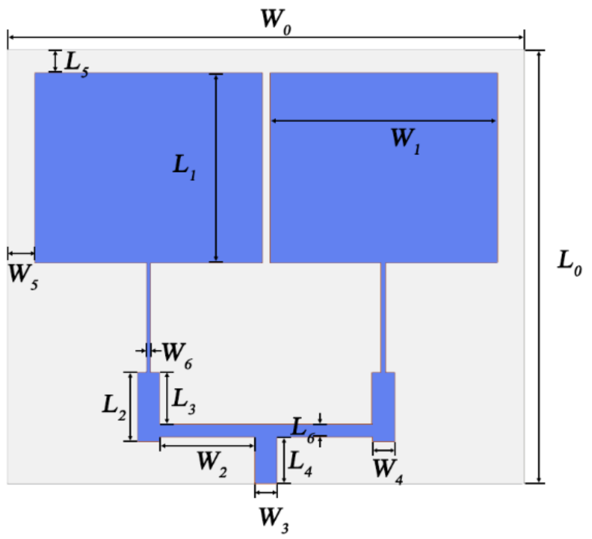

Fig. S6 The proposed 1 × 2 patch antenna array. The detailed dimensions of the four antennas are as follows: W_0 = 227.25 mm/69.00 mm/62.385 mm/55.00 mm, W_1 = 72.6 mm/29.00 mm/26.8 mm/24.2 mm, W_2 = 30.465 mm/12.18 mm/11.285 mm/10.155 mm, W_3 = 7.11 mm/2.84 mm/2.63 mm/2.37 mm, W_4 = 38.16 mm/4.32 mm/3.26 mm/2.345 mm, W_5 = 8.7 mm/3.5 mm/3.25 mm/2.9 mm, W_6 = 1.32 mm/0.53 mm/0.49 mm/0.415 mm, L_0 = 138.42 mm/55.355 mm/51.24 mm/46.14 mm, L_1 = 60.75 mm/24.24 mm/22.5 mm/20.25 mm, L_2 = 22.11 mm/8.84 mm/8.19 mm/7.37 mm, L_3 = 15.00 mm/6.00 mm/5.56 mm/5.505 mm, L_4 = 15.00 mm/6.00 mm/5.56 mm/5.00 mm, L_5 = 7.125 mm/2.88 mm/2.65 mm/5.275 mm, L_6 = 4.08 mm/1.63 mm/1.51 mm/1.36 mm. The thickness of copper radiation patch and ground plate is 0.018 mm

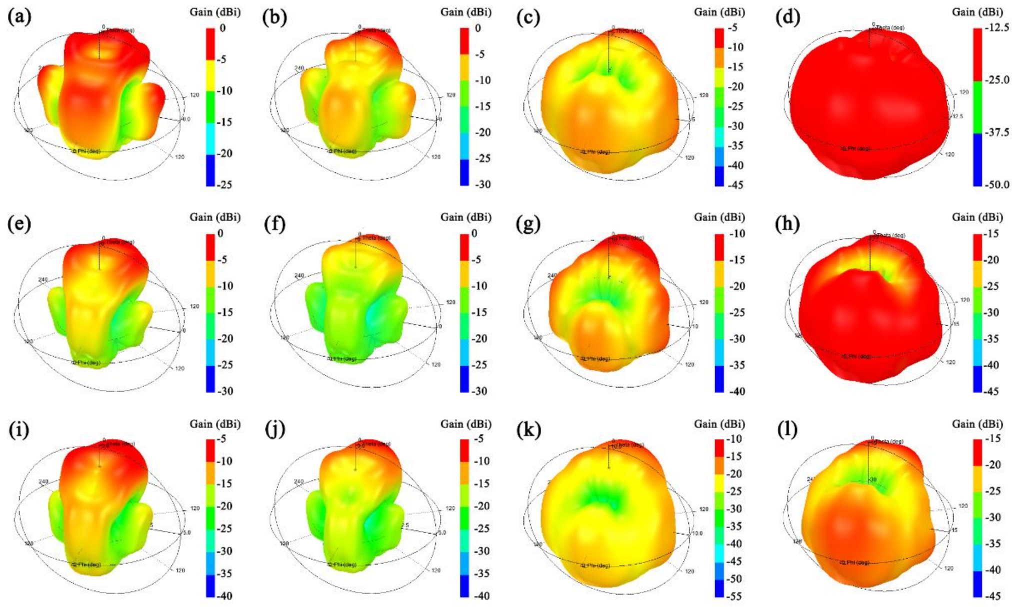

Fig. S7 The 3D radiation patterns of **a** MT-5, **b** MT-4, **c** MT-3, **d** MT-2 antenna with a substrate thickness of 0.4 mm. The 3D radiation patterns of **e** MT-5, **f** MT-4, **g** MT-3, **h** MT-2 antenna with a substrate thickness of 0.7 mm. The 3D radiation patterns of **i** MT-5, **j** MT-4, **k** MT-3, **l** MT-2 antenna with a substrate thickness of 1.0 mm

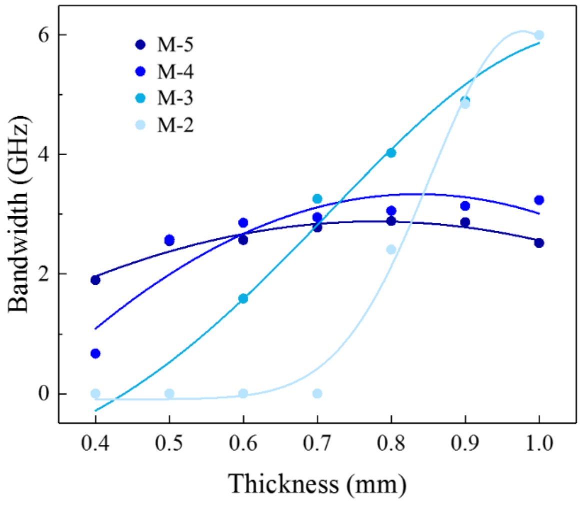

Fig. S8 The bandwidth of four antennas with different substrate thicknesses

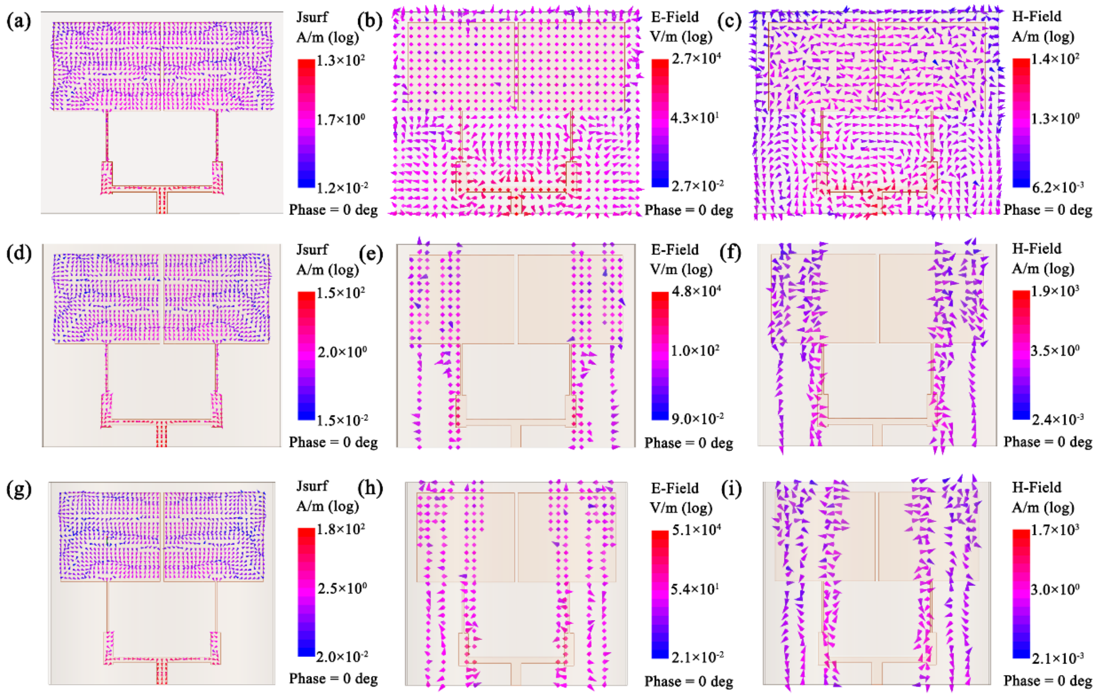

Fig. S9 **a** The surface current distribution, **b** E-field distribution, and **c** H-field distribution of unbending antenna at 15 GHz. **d** The surface current distribution, **e** E-field distribution, and **f** H-field distribution of bending antenna with R = 80 mm at 15 GHz. **g** The surface current distribution, **h** E-field distribution, and **i** H-field distribution of bending antenna with R = 40 mm at 15 GHz

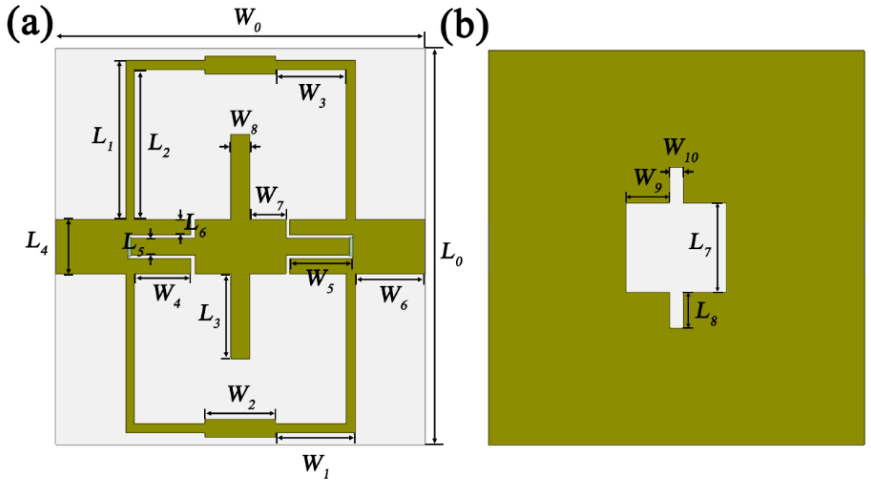

Fig. S10 **a** The top view and **b** bottom view of the UWB bandpass filter. The detailed dimensions of the bandpass filter are as follows: W_0 = 10.5 mm, W_1 = 2.26 mm, W_2 = 2.00 mm, W_3 = 2.01 mm, W_4 = 1.61 mm, W_5 = 1.80 mm, W_6 = 1.61 mm, W_7 = 1.03 mm, W_8 = 0.54 mm, W_9 = 1.21 mm, W_{10} = 0.38 mm, L_0 = 11.00 mm, L_1 = 4.42 mm, L_2 = 4.17 mm, L_3 = 2.37 mm, L_4 = 1.50 mm, L_5 = 0.51 mm, L_6 = 0.41 mm, L_7 = 2.50 mm, L_8 = 1.00 mm. The thickness of copper radiation patch and ground plate is 0.035 mm

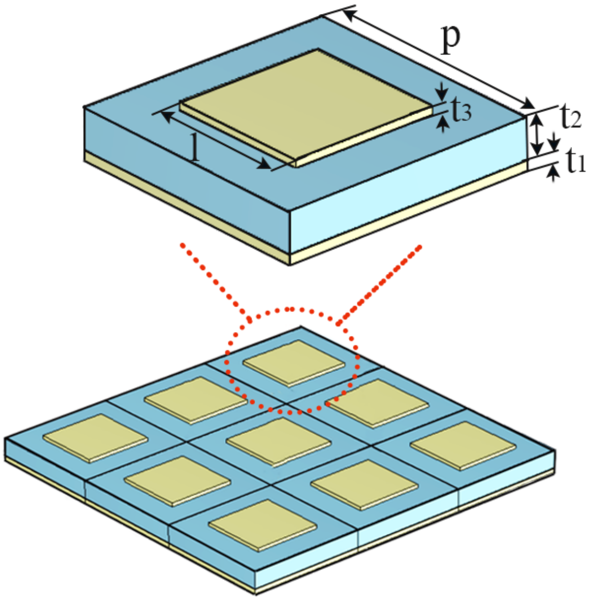

Fig. S11 The detailed dimensions of the infrared stealth device are as follows: p= 1000 nm, l =570 nm, t_1 = 40 nm, t_2 = 150 nm, t_3 =30 nm

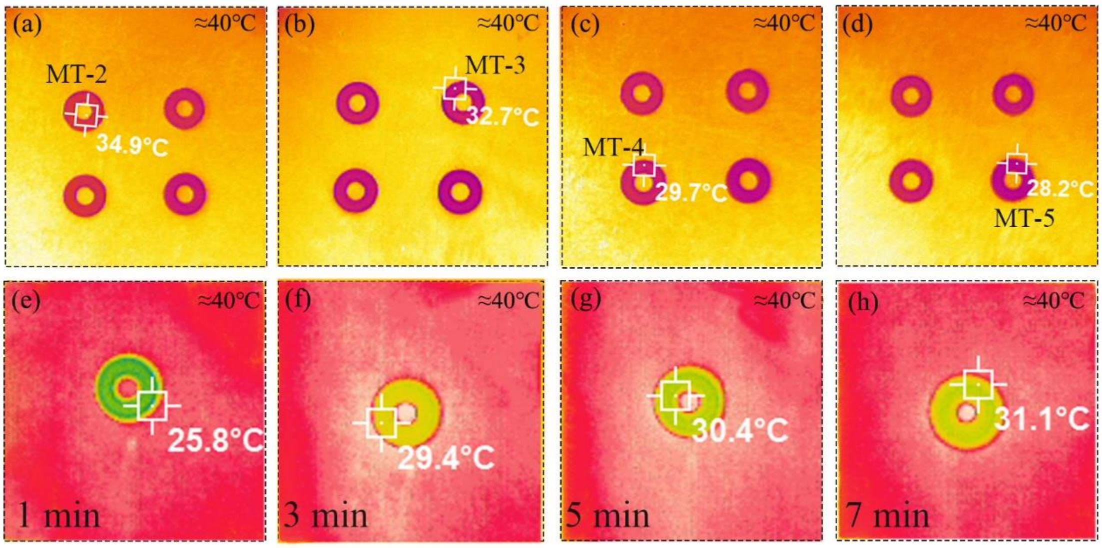

Fig. S12 a-d Thermal infrared images of MXene/TiO_2 hybrids with different calcination temperatures captured at 2 min. Thermal infrared images of MT-5 captured at **e** 1, **f** 3, **g** 5, **h** 7 min, respectively.

Table S1 The key parameters of previous reported the same-type electromagnetic device

Materials	Antennas		Filter		Infrared stealth	
	S_{11} (dB)	effective bandwidth (<-10dB)	Passband bandwidth	suppression outside the band	Minimum emissivity	effective bandwidth
MXene [S1]	-48	3.25GHz				
$Ti_3C_2T_x$ [S2]	−24.25	0.06GHz				
Graphene Microlaminates [S3]	-51	1.6				
MXene-SWNT [S4]	-36dB	0.35GHz				
Graphene nanoplates [S5]			0.75	-62.5dB		
Graphene flakes [S6]			0.25	-57		
Graphene-assembled film [S7]			1	-42		
ITO/dielectric/ITO sandwiched structure [S8]					0.52	2.5 GHz
Metasurface [S9]					0.2	8-14 μm
Ceramic substrate [S10]					0.22	3-14 μm
Hybrid metasurface [S11]					0.32	3-14 μm
PTFE top-covered multi-layer composite structure [S12]					0.196	8–14 μm
FeAl mixture [S13]					0.15	8–14 μm
Carbon nanotube film [S14]	-37.8	0.752				
Carbon nanotube film [S14]	-25.9	0.67				
Carbon nanotube film [S 14]	-35.6	0.698				
This work	-63.2	2.7	5.44	53.4	0.027	6-14 μm

Table S2 Minimum and average emissivity of MXene with different calcination temperatures

Sample	MT-2	MT-3	MT-4	MT-5
Minimum emissivity	0.036	0.035	0.032	0.027
Average emissivity	0.188	0.152	0.124	0.077

Table S3 Average visible light absorptivity of MXene with different calcination temperatures

Sample	MT-2	MT-3	MT-4	MT-5
Average absorptivity	36.3	54.5	57.7	78.2

Supplementary References

[S1] M.K. Han, Y.Q. Liu, R. Rakhmanov, C. Israel, M.A. Tajin et al., Solution-processed $Ti_3C_2T_x$ MXene antennas for radio-frequency communication. Adv. Mater. **33**(1), 2003225 (2021). https://doi.org/10.1002/adma.202003225

[S2] K.K. Kazemi, S.J. Hu, O. Niksan, K.K. Adhikari, N.R. Tanguy et al.,, Low-profile planar antenna sensor based on $Ti_3C_2T_x$ MXene membrane for VOC and humidity monitoring. Adv. Mater. Interfaces **9**(13), 2102411 (2022). https://doi.org/10.1002/admi.202102411

[S3] J.C. Shu, M.S. Cao, Y.L. Zhang, Y.Z. Wang, Q.L. Zhao et al., Atomic-molecular

engineering tailoring graphene microlaminates to tune multifunctional antennas. Adv. Funct. Mater. **33**(15), 2212379 (2023). https://doi.org/10.1002/adfm.202212379

[S4] Y. Li, X. Tian, S.P. Gao, L. Jing, K.r. Li et al., Reversible crumpling of 2D titanium carbide (MXene) nanocoatings for stretchable electromagnetic shielding and wearable wireless communication. Adv. Funct. Mater. **30**(5), 1907451 (2020). https://doi.org/10.1002/adfm.201907451

[S5] L. Li, Y.X. Cheng, J.Z. Chen, R. Hou, T. Su, Attenuation-tunable balanced bandpass filter based on graphene nanoplates. Int. J. RF Microw. Comput. Aided Eng. **32**(12), 23511 (2022). https://doi.org/10.1002/mmce. 23511

[S6] J.Z. Chen, S.Y. Zhu, L. Li, C. Fan, Microstrip bandpass diplexer with linear-tunable attenuation based on graphene flakes. Mater. Lett. **316**, 132059 (2022). https://doi.org/10.1016/j.matlet.2022.132059

[S7] B.Q. Huang, S.T. Li, R.G. Song, Z.Y. Hou, C.G. Liu, D.P. He, High-conductivity graphene-assembled film-based bandpass filter for 5G applications. Int. J. RF Microw. Comput. Aided Eng. **31**, 22602 (2021). https://doi.org/10.1002/mmce.22602

[S8] C.L. Xu, B.K. Wang, M.B. Yan, Y.Q. Pang, Y.Y. Meng et al., An optically transparent sandwich structure for radar-infrared bi-stealth. Infrared Phys. Techn. **105**, 103108 (2020). https://doi.org/10.1016/j.infrared.2019.103108

[S9] C. Zhang, J. Yang, W. Yuan, J. Zhao, J.Y. Dai et al., An ultralight and thin metasurface for radar infrared bi-stealth applications. J. Phys. D: Appl. Phys. **50**(44), 444002 (2017). https://doi.org/10.1088/1361-6463/aa8ba6

[S10] W.J. Wang, J.M. Jiang, J.G. Liang, Z.X. Wang, C.L. Xu et al., A multifunctional coating for radar-infrared stealth-compatible at high temperatures. IEEE Access **10**, 122280-122285 (2022). https://doi.org/10.1109/ACCESS.2022.3223442

[S11] C.L. Xu, B.K. Wang, Y.Q. Pang, J.F. Wang, M.B. Yan et al., Hybrid metasurfaces for infrared-multiband radar stealth-compatible materials applications. IEEE Access **7**, 147586-147595 (2019). https://doi.org/10.1109/ACCESS.2019.2946405

[S12] D. Qi, Y.Z. Cheng, X. Wang, F. Wang, B.W. Li, R.Z. Gong, Multi-layer composite structure covered polytetrafluoroethylene for visible-infrared-radar spectral compatibility. J. Phys. D: Appl. Phys. **50**, 505108 (2017). https://doi.org/10.1088/1361-6463/aa95a9

[S13] H.L. Lv, G.B. Ji, X.G. Li, X.F. Chang, M. Wang et al., Microwave absorbing properties and enhanced infrared reflectance of FeAl mixture synthesized by two-step ball-milling method. J. Magn. Magn. Mater. **374**, 225–229 (2015). http://dx.doi.org/10.1016/j.jmmm.2014.08.006

[S14] H. Song, H. Jeon, D. Im, N. Çakmakçı, K.Y. Shin, Y. Jeong, Free-standing carbon nanotube film for high efficiency monopole antenna. Carbon **187**, 22-28 (2022). https://doi.org/10.1016/j.carbon.2021.10.068
